# Comparison Of The Gut Microbiota In Different Age Groups In China

**DOI:** 10.3389/fcimb.2022.877914

**Published:** 2022-07-25

**Authors:** Hang Yan, Qian Qin, Su Yan, Jingfeng Chen, Yang Yang, Tiantian Li, Xinxin Gao, Suying Ding

**Affiliations:** ^1^ Health Management Center, The First Affiliated Hospital of Zhengzhou University, Zhengzhou, China; ^2^ College of Public Health, Zhengzhou University, Zhengzhou, China

**Keywords:** aging, 50 years old, gut microbiota, metagenomics, metabolic pathways

## Abstract

Aging is now the most profound risk factor for almost all non-communicable diseases. Studies have shown that probiotics play a specific role in fighting aging. We used metagenomic sequencing to study the changes in gut microbes in different age groups and found that aging had the most significant effect on subjects’ gut microbe structure. Our study divided the subjects (n=614) into two groups by using 50 years as the age cut-off point for the grouping. Compared with the younger group, several species with altered abundance and specific functional pathways were found in the older group. At the species level, the abundance of *Bacteroides fragilis*, *Bifidobacterium longum*, *Clostridium bolteae*, *Escherichia coli*, *Klebsiella pneumoniae*, and *Parabacteroides merdae* were increased in older individuals. They were positively correlated to the pathways responsible for lipopolysaccharide (LPS) biosynthesis and the degradation of short-chain fatty acids (SCFAs). On the contrary, the levels of *Barnesiella intestinihominis*, *Megamonas funiformis*, and *Subdoligranulum unclassified* were decreased in the older group, which negatively correlated with the above pathways (p-value<0.05). Functional prediction revealed 92 metabolic pathways enriched in the older group significantly higher than those in the younger group (p-value<0.05), especially pathways related to LPS biosynthesis and the degradation of SCFAs. Additionally, we established a simple non-invasive model of aging, nine species (*Bacteroides fragilis*, *Barnesiella intestinihominis*, *Bifidobacterium longum*, *Clostridium bolteae*, *Escherichia coli*, *Klebsiella pneumoniae*, *Megamonas funiformis*, *Parabacteroides merdae*, and *Subdoligranulum unclassified*) were selected to construct the model. The area under the receiver operating curve (AUC) of the model implied that supplemented probiotics might influence aging. We discuss the features of the aging microbiota that make it more amenable to pre-and probiotic interventions. We speculate these metabolic pathways of gut microbiota can be associated with the immune status and inflammation of older adults. Health interventions that promote a diverse microbiome could influence the health of older adults.

## Introduction

Aging was defined as a process of comprehensive decline in physiological functions caused by genetic determination and environmental regulation (Mangiola et al., 2018), which was related to immune homeostasis, free radicals, mitochondrial DNA damage, telomerase, and silent information regulator genes (Lopez-Otin et al., 2013). In addition, gut microbiota was at the core of many age-related changes and played an essential role in the lifespan of different species (Bana and Cabreiro, 2019; Badal et al., 2020). Compared with young mice, the composition of the gut microbes of old mice has changed: α diversity increases, the ratio of *Firmicutes/Bacteroides phylum* increases (Hoffman et al., 2017). In addition, Rondanelli et al. (Rondanelli et al., 2015) mentioned that the abundance of *Bifidobacteria*, *Firmicutes*, *Enterococcus faecalis*, and *Enterobacter pseudococcus* were reduced, and the levels of *Bacteroides* and *Enterobacteriaceae* increased in the elderly group when contrasted with the younger group. Age-related changes in the composition of the microbiota have been shown to play a significant part in developing neurodegenerative diseases such as Parkinson’s disease and Alzheimer’s disease (Pistollato et al., 2016; Sampson et al., 2016; Cattaneo et al., 2017). Furthermore, the considerable life extension effect of supplementing probiotics was found in Caenorhabditis elegans, which was considered to be related to the improvement of resistance to oxidative stress and innate immune response (Kwon et al., 2016; Nakagawa et al., 2016). When gut microbes from old mice were transplanted into young mice *via* fecal bacterial transplantation, splenic CD4+ T cell differentiation and the circulation of bacterial-derived inflammatory factors were increased, intestinal inflammation and the expression of inflammatory cytokine genes (such as tumor necrosis factor-α) were up-regulated (Fransen et al., 2017). This was significantly improved by transplanting normal mouse fecal bacteria or supplementing with *Akkermansia muciniphila*, demonstrating the critical role of gut microbial imbalances in aging and premature death in mice (Barcena et al., 2019). The gut microbiota communicates with the host through various biomolecules and nutrient signaling-independent pathways. Disturbance of these communications by age-related gut dysbiosis can affect the host’s health and lifespan. This may explain the relationship between gut microbiota and aging.

Studies have shown that chronic low-grade inflammation (Perez Martinez et al., 2014; Lowry et al., 2016; DeJong et al., 2020) and immune dysfunction (Kim and Jazwinski, 2018) were the main characteristics of aging. The gut microbes participated in developing the intestinal mucosal immune system, regulating immune homeostasis and inflammation (Konturek et al., 2015). The Th1 and Th17 cells in the sterile mice were significantly reduced, but they were recovered after rebuilding the gut microbes (Barcena et al., 2019). The analysis of bacterial metabolites revealed that the increased degradation of short-chain fatty acids (SCFAs) and production of lipopolysaccharides (LPS) could promote increased intestinal permeability and aggravate the intestinal itself and systemic inflammation. The above indicated the mechanism of gut microbes in aging (Keebaugh and Ja, 2017; Haran and McCormick, 2021).

The current research results on the aging and gut microbiome were heterogeneous. Different age groups had different results. Most elderly samples were defined as including those over 65 years old or over 60 years old (Badal et al., 2020). Still, our study considered the influence of sex hormones on the gut microbiome and used 50 years as the cut-off point to study gut microbes changes in the younger and older groups. We used metagenomic sequencing to study gut microbiome changes across age groups in 614 adults (19-87 years). We analyzed the eating habits and laws, smoking and drinking, glucose and lipid metabolism, related medical history of these subjects and found that aging was the most important cause of changes in the gut microbiome. These findings suggested that the gut microbiome may become a new target and provide fresh ideas and directions for delaying aging.

## Materials and Methods

### Study Population

For this study, we selected 614 subjects who underwent routine examinations and metagenomic sequencing of fecal microbiota at the Health Management Center of the First Affiliated Hospital of Zhengzhou University from May 2018 to May 2019. The studies involving human participants were reviewed and approved by an ethics committee from the First Affiliated Hospital of Zhengzhou University (Approval number: 2018-KY-56). The subjects were divided into the younger group (19<age<50) and the older group (50≤age<87) (Dabbs, 1990; Org et al., 2016; He et al., 2019; Zhao et al., 2019). There were 372 cases in the younger group, with an average age of 37.48 ± 7.14 years, and 242 patients in the older group, with an average age of 58.09 ± 6.85 years. Exclusion criteria: Subjects with diabetes, hypertension, coronary atherosclerotic heart disease, hyperthyroidism, hypothyroidism, Cushing syndrome, and so on; Subjects have taken antibiotics, probiotics, cholesterol-lowering drugs, hormone drugs (estrogen, glucocorticoid, etc.) in the past three months; Subjects with major malignant diseases (tumor, etc.); Subjects with incomplete laboratory test results or incomplete questionnaires.

### Basic Information and Biochemical Indicators

The full-time staff guided the subjects one-on-one to fill out questionnaires, including disease history, medication history, diet and exercise habits, etc. Waist circumference (WC) (Separate feet with the same shoulder width and use a soft ruler to wrap around the umbilicus at the end of the exhalation), weight, and height (Computerized body scale, SK-X80) were estimated. Body mass index (BMI) (Kg/m^2^) was calculated as weight/height^2^. Blood pressure (OMRON Medical automatic electronic blood pressure monitor, HBP-9021) was scaled simultaneously. Fasting venous blood (8-10h) was extracted overnight, and serum was separated within 2 hours. Serum test results [fasting blood glucose (FBG), low-density lipoprotein cholesterol (LDL), high-density lipoprotein cholesterol (HDL), triglyceride (TG), and total cholesterol (TC)] of the participants were obtained from hospital laboratories. On the same day, fecal samples were collected and separately packed and then placed in a -80°C refrigerator for later testing.

### DNA Extraction, Shotgun Metagenomic Sequencing, and Quantity Control of Reads

According to the manufacturer’s instructions, DNA was extracted from a total of 614 stool samples using the MagPure Stool DNA KF kits. Angen Biotech Co., Ltd (Yayingshi Road, Luogang District, Guangzhou, China) provided the DNA extraction kit. We used a unique stool collection tube (Sarstedt stool collection tube, Germany) to collect the samples. It was immediately cooled and stored at80 degrees after the stool was collected. Take 0.2 mg of frozen feces for DNA extraction. The DNA library construction based upon the DNA nanospheres (DNB) and the shotgun metagenomic sequencing-based upon the combined probe anchoring synthesis (CPAs) was carried out for all samples (MGI2000, MGI, Shenzhen, China). The all-inclusive accuracy (OA≥0.8) control strategy detailed above was used to perform quality control (QC) on the original reads to filter out the low-quality reads (Fang et al., 2018).

1ug DNA was used to construct libraries. Firstly, DNA was randomly fragmented by Covairs (Covaris M220, Covaris, American). Secondly, the average size of 200-400bp fragments Magnetic beads selected DNA, end-repair 3’ adenylated, adapters-ligation, PCR Amplifying and using Magnetic beads to purify the products. Thirdly, double stranded DNA was denatured into single stranded DNA after heating and annealed to form single strand circle DNA by y the splint oligo sequence. The single-strand circle DNA were formatted as the final library and qualified by QC. The qualified libraries were sequenced on the MGISEQ-2000 platform (BGI-Shenzhen, China). The sequencing length was pair-end 100bp, and the average sequencing data of each sample was about 10Gb raw data. The fastqc software was used to check the sequencing reads quality. The filter standard: filter the reads whose base quality value is lower than 20; filter the reads containing adapter; filter the reads containing N bases more than 5%.

### Microbiome Composition and Function Profiling

Standard relative abundance values of all species at all levels were obtained by MetaPhlAn2, which was used to carry out the metagenomic classification of sequenced libraries. Firstly, it contrasted between sequence and marker. The MetaPhlAn2 (Truong et al., 2015) classifier lastly distinguished metagenomic reads against the precomputed marker catalog using nucleotide BLAST (Priyam et al., 2019) searches to provide abundances in clade for either one or more sequenced metagenomes. Secondly, the total number of reads in each clade was normalized by the classifier through the nucleotide length of its markers. The relative abundance of each taxonomic unit was calculated, considering any titles specific to subclades. Therefore, the microbial clade anomaly was estimated by normalizing read-based counts by the average genome size of each clade. Using these methods, we generated a map of the gut microbes containing bacteria. Non-redundant gene sets were annotated using the NCBICOG database (version 2014). Functional genes were annotated to the KEGG metabolic pathway using humannV1.0.0 and further annotated at the KEGG metabolic module (module) level to obtain the composition of the metabolic pathway finally (HMP Unified Metabolic Analysis Network 2) (Fang et al., 2018; Franzosa et al., 2018).

### Statistical Analysis

R procedure (version 4.0.2) was used to perform statistical analysis. Standardized statistical methods were used for analyzing laboratory test results and demographic information. Continuous variables were presented as mean ± standard deviation ( $\bar{\text{x}}$ ±s), while classified variables were shown as counts. Above all, normality and homogeneity of variance were tested for the difference analysis between groups and p-value≥0.05 was known as normality and homogeneity of variance. Furthermore, parametric test (t-test) or nonparametric test (rank-sum test) was used as statistical analysis. The chi-square test was used to determine differences in constituent ratios between groups. Spearman correlation analysis was used to assess the correlation between differential microbiota and covariates. A permutational multivariate analysis of variance (PERMANOVA) and redundancy analysis (RDA) was performed to verify whether aging was the most significant influencing factor. The Shannon, obs, bray, and Spearman indexes of each sample were calculated by the “vegan” package in R. Using the R program “ade4” for visual analysis to perform Principal coordinate analysis (PCoA) (Jari Oksanen et al., 2020). We used STAMP (version 2.1.3) to analyze the differences in the microbiota at the phylum through species levels and pathways. We used white’s nonparametric test to calculate the difference between groups and Benjamini-Hochberg FDR for multiple test correction. We dismissed species with low occurrences (positivity rates <10%) before analyzing the differential microbiotas, p-value<0.05 was considered statistically significant. Finally, we used logistic regression following the different analysis results to establish a prediction model. The samples were divided into training and test sets. A logistic regression model was based on the training set, and verification was carried out in the substitution test set. A receiver operating characteristic (ROC) curve was used to compare the effectiveness of prediction models (Qin et al., 2021).

## Results

### Clinical Characteristics of Subjects

Six hundred fourteen subjects were included, including 372 subjects in the younger group (mean age 37.48 ± 7.14 years) and 242 subjects in the older group (mean age 58.09 ± 6.85 years). The levels of age, BMI, WC, SBP, DBP, FBG, TC, LDL, diet habits, and wholegrain in the older group were significantly higher than those in the younger group (p-value<0.05). There were no significant differences in gender, TG, HDL, diet regular, sport, smoking, and drinking between the two groups (**Table 1**).

**Table 1:** The essential characteristics and laboratory test results.

	Younger	Older	t/χ2	p-value
gender	male229; female143	male130; female112	3.71	0.054
age	37.48 ± 7.14	58.09 ± 6.85	-35.53	<0.001*
BMI	24.38 ± 3.55	25.22 ± 2.99	-3.03	0.003*
WC	83.26 ± 10.58	86.64 ± 9.48	-4.03	<0.001*
DBP	75.26 ± 11.18	79.56 ± 12.42	-4.46	<0.001*
SBP	122.91 ± 14.73	131.47 ± 18.47	-6.36	<0.001*
FBG	5.19 ± 1.08	5.79 ± 1.48	-5.72	<0.001*
TC	4.62 ± 0.84	4.99 ± 0.9	-5.11	<0.001*
TG	1.48 ± 1.34	1.66 ± 1	-1.72	0.086
HDL	1.42 ± 0.36	1.47 ± 0.39	-1.4	0.163
LDL	2.79 ± 0.75	3.08 ± 0.82	-4.54	<0.001*
sport	not:48, rarely:182, frequently:142	not:40, rarely:109, frequently:93	1.81	0.404
Diet regular	Y325; N47	Y221; N21	2.33	0.127
Diet habit	mix281; meatarian37; vegetarian54	mix173; meatarian13; vegetarian56	10.18	0.006*
wholegrain	Y278; N94	Y202; N40	6.56	0.01*
smoking	Y84; N288	Y43; N199	2.07	0.15
drinking	Y142; N230	Y89; N153	0.12	0.727

WC: waist circumference; BMI: body mass index; SBP: systolic blood pressure; DBP: diastolic blood pressure; FBG: fasting blood glucose; TC: total cholesterol; TG: triglyceride; HDL: high-density lipoprotein; LDL: low-density lipoprotein cholesterol. Student’s t-tests were used to compare the differences between the younger group (n=372) and the older group (n=242). *p-value<0.05.

### Analysis of Factors Influencing the Gut Microbes

**Figure 1:**
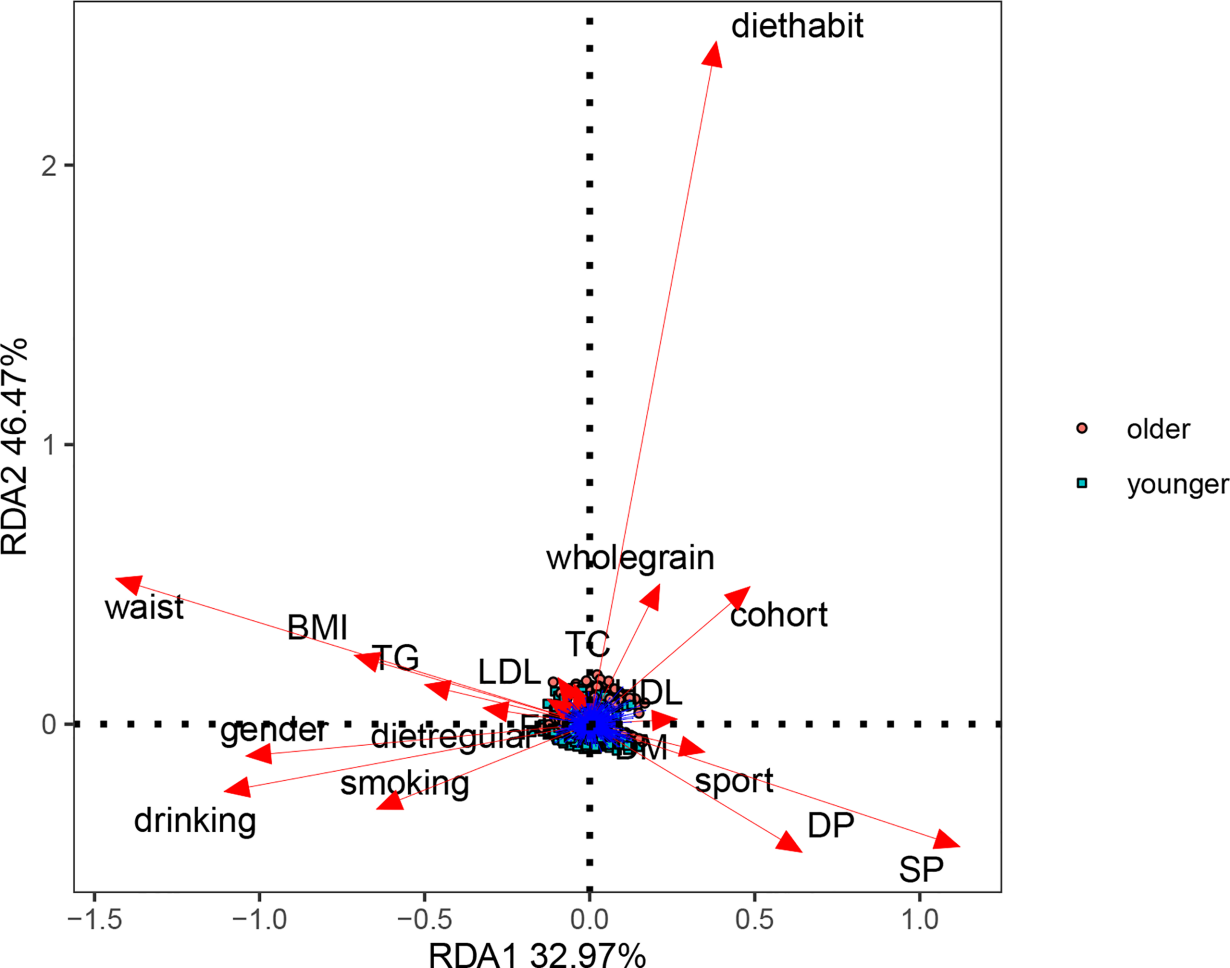
Effects of dietary habits and individual attributes on microbiome.

We analyzed the basic information of the included population (i.e., age, gender, BMI, waist circumference, blood pressure, blood sugar, blood lipids, smoking, drinking, regular diet, eating habits, whole-wheat consumption, exercise, and chronic diseases) by PERMANOVA. In univariate and multivariate analyses, aging had the most significant effect on participants’ gut microbe structure (p-value<0.05, **Table 2**). We constructed an RDA diagram to reflect the relationship between the microbiome and participants’ dietary habits and individual attributes (**Figure 1**). In this result, we found that diethabit is one of the most influential environmental factors, diethabit, wholegrain, TC, HDL, sport, DP, and SP all positively correlated with the cohort, suggesting that these factors were closely related to the changes of the gut microbiome.

**Table 2:** The influence of the basic attributes of the participants on the gut microbiome.

Phenotype	single factor			multifactor		
	F.Model	Variation (R^2^)	p-value	F.Model	Variation (R^2^)	p-value
cohort	3.612	0.006	0.001*	3.605	0.006	0.001*
Gender	0.754	0.001	0.713	0.801	0.001	0.649
BMI	0.892	0.001	0.546	0.625	0.001	0.864
DP	0.928	0.002	0.485	0.877	0.001	0.568
SP	0.618	0.001	0.857	1.352	0.002	0.143
Waist	1.224	0.002	0.199	1.152	0.002	0.288
Regular meals	1.284	0.002	0.177	1.088	0.002	0.338
Dietary habit	0.575	0.001	0.9	0.635	0.001	0.848
Wholegrains	0.948	0.002	0.468	0.869	0.001	0.576
Drinking	0.737	0.001	0.727	0.686	0.001	0.809
Smoking	1.243	0.002	0.21	1.068	0.002	0.354
FBG	2.597	0.004	0.008*	2.419	0.004	0.016*
TC	1.056	0.002	0.332	0.989	0.002	0.406
TG	0.598	0.001	0.888	0.376	0.001	0.991
HDL	0.627	0.001	0.845	1.234	0.002	0.214
LDL	1.426	0.002	0.112	1.084	0.002	0.346
Sport	1.006	0.002	0.371	1.075	0.002	0.325

^*^p-value<0.05.

### Analysis of Microbiota Diversity

**Figure 2:**
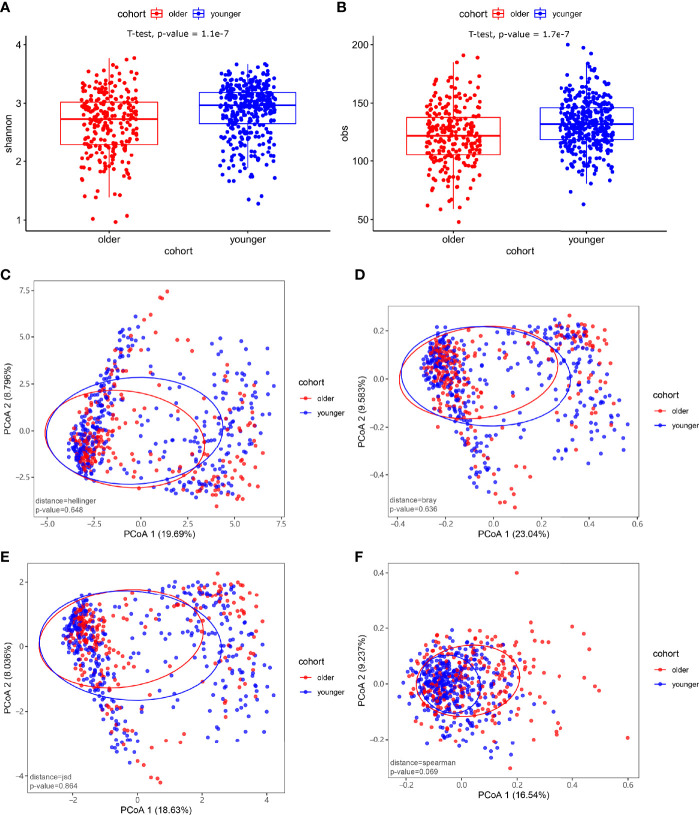
Microbiome composition and diversity. **(A, B)** Alpha diversity was measured by the Shannon and obs indices for comparisons between the two groups. There were significant differences in the level of microbial species between the younger and older groups (p-value<0.05). **(C–F)** Beta diversity between the younger and older groups. Beta diversity was calculated based on the Hellinger distance, JSD distance, Bray distance, and Spearmen distance. There were no significant differences between the two groups.

At the species level, significant differences between the microbiomes of the younger and older subjects were observed in alpha diversity (p-value<0.05) (**Figures 2A, B**). There were no significant differences in the species-level beta diversity between the younger and older groups (p-value=0.648, p-value=0.636, p-value=0.864, and p-value=0.069, respectively, **Figures 2C–F**).

### The Gut Microbiome Characteristics in Different Groups

**Figure 3:**
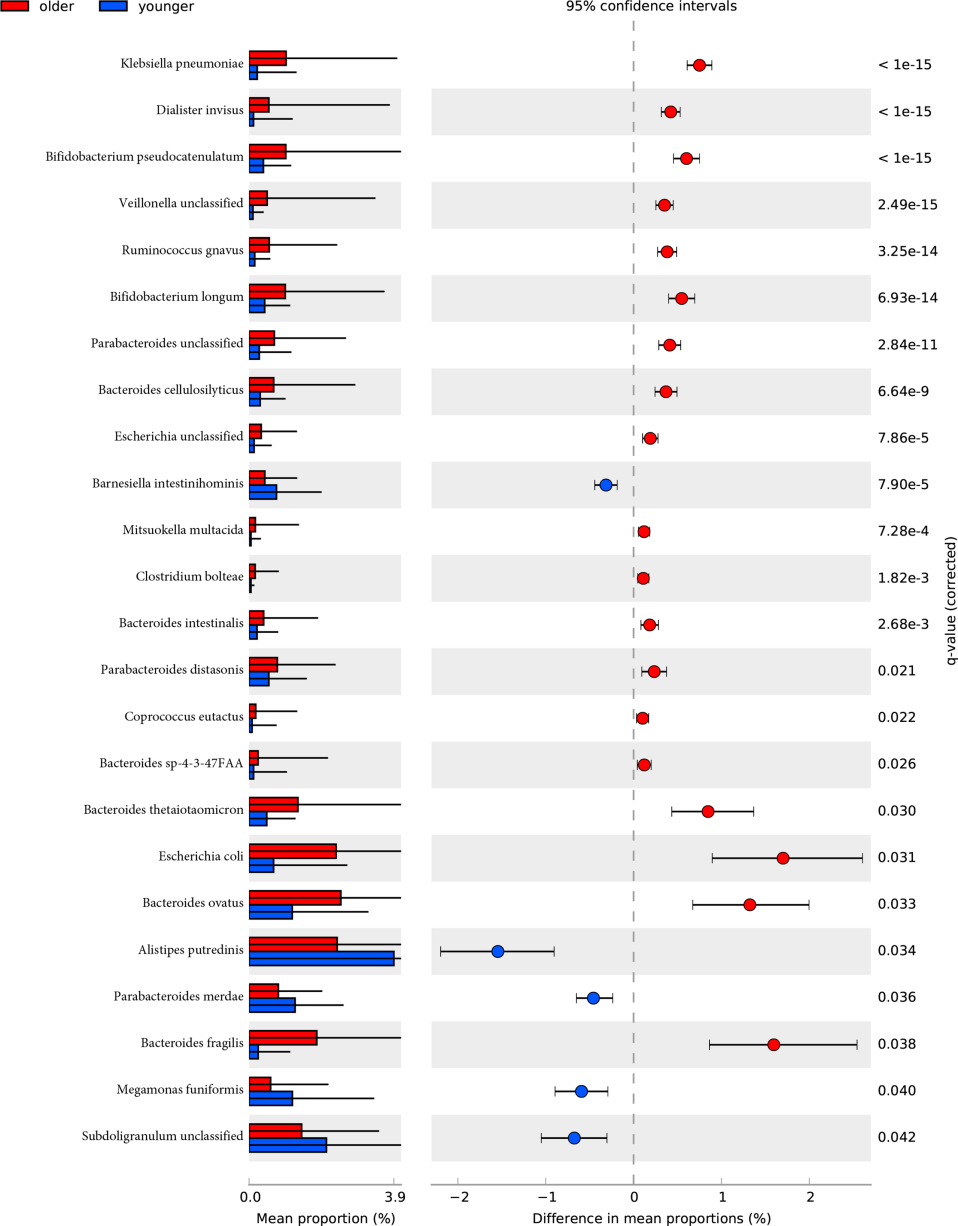
The relative abundance of bacterial species between younger and older groups. Wilcoxon tests analysis of the relative abundance of bacterial species showed significant differences in 24 species, with five species enriched in the younger group and 19 enriched in the older group.

The species-level analysis found that 24 species in the older group were significantly different in abundance than the younger group after removing the species with low incidence and abundance (p-value<0.05). Five were enriched in the younger group, and 19 were enriched in the older group. Wilcoxon test showed that the younger group enriched the following bacteria: *Alistipes putredinis*, *Barnesiella intestinihominis*, *Megamonas funiformis*, *Parabacteroides merdae*, *Subdoligranulum unclassified*. We found that *Bacteroides* (*Bacteroides cellulosilyticus, Bacteroides fragilis, Bacteroides intestinalis, Bacteroides ovatus, Bacteroides sp 4 3 47FAA, Bacteroides thetaiotaomicron*), *Bifidobacterium* (*Bifidobacterium longum, Bifidobacterium pseudocatenulatum*), *Clostridium bolteae*, *Escherichia* (*Escherichia coli, Escherichia unclassified*), *Parabacteroides* (*Parabacteroides distasonis, Parabacteroides unclassified*), *Ruminococcus gnavus*, *Klebsiella pneumoniae*, *Dialister invisus*, *Veillonella unclassified*, *Mitsuokella multacidawere* and *Coprococcus eutactus* were enriched in the older group (**Figure 3**).

### Functional Shifts in the Microbiome Characteristics of Different Groups

**Figure 4:**
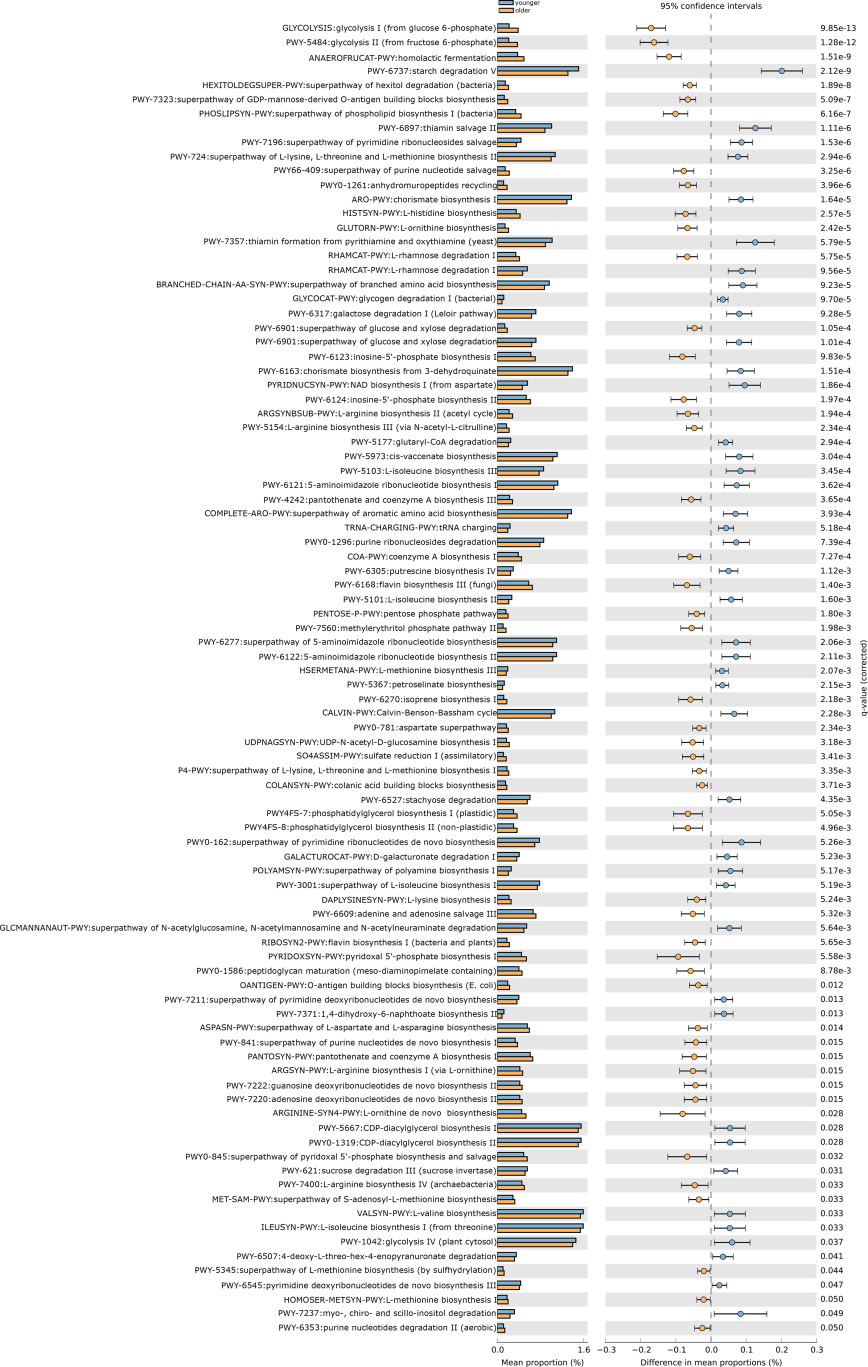
The functional shifts of bacterial species between younger and older groups.

After removing low abundance pathways, we constructed functional profiles for each sample using 92 microbial MetaCyc pathways. They are significantly different between the younger and older groups (**Figure 3**). Forty-eight of which were enriched in the older subjects. Among the 48 pathways, PWY-7323 and OANTIGEN-PWY were involved in the production of LPS, ANAEROFRUCAT-PWY was included in the degradation of SCFAs; 4 pathways (PWY-6901, RHAMCAT-PWY, COLANSYN-PWY, and UDPNAGSYN-PWY) were responsible for the generation or degradation of carbohydrates. PWY4FS-8 and PHOSLIPSIN-PWY were accountable for the generation of fatty acids and lipids. 22.91% of pathways were responsible for the production of amino acids. 14.58% of pathways were responsible for the biosynthesis of nucleotides and nucleotides. Other pathways were responsible for the biosynthesis and degradation of secondary metabolites, the metabolism of inorganic nutrients, cofactors, carriers, vitamin biosynthesis, etc. 44 pathways were enriched in the younger group. 22.72% of pathways were in charge of the biosynthesis of amino acids. PWY-5367, PWY-6277, PWY-5973, and PWY0-1319 were responsible for the biosynthesis of fatty acids and lipids. 15.90% of pathways were in charge of the biosynthesis and degradation of nucleosides and nucleotides. 13.63% of pathways were responsible for carbohydrate degradation, sugar biosynthesis, or glycogen degradation. Other pathways were responsible for aromatic compound biosynthesis, cofactors, carrier, and vitamin biosynthesis, amine and polyamine biosynthesis, arginine and polyamine biosynthesis, and so on (**Figure 4**).

Ninety-two pathways were significantly different between the two groups, of which 48 pathways were enriched in the older group, and 44 pathways were enriched in the older group.

### Relationship Between Functional Shifts and Microbiome Characteristics

**Figure 5:**
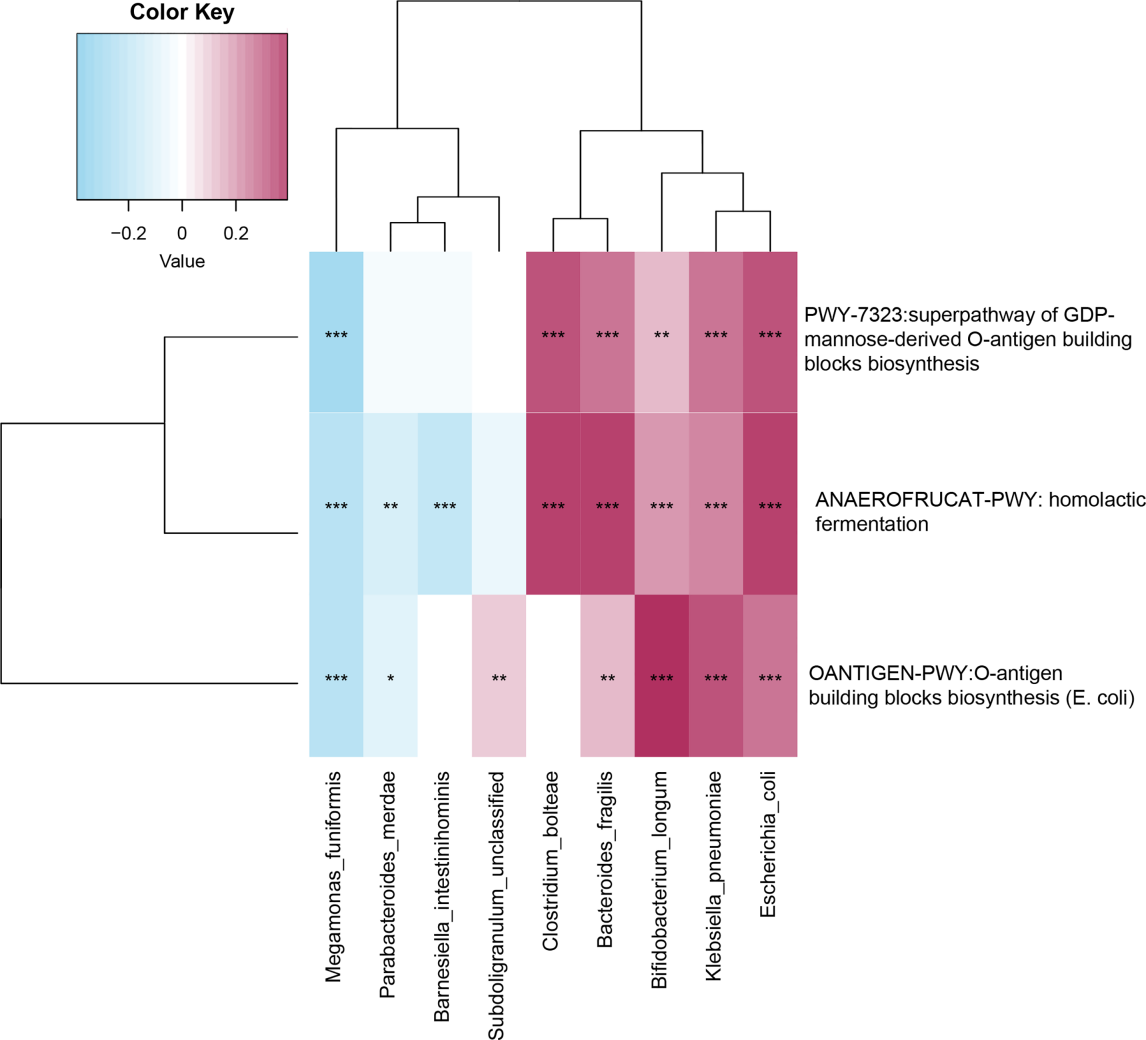
Spearman’s correlation matrix for microbial pathways and species. Blue signifies a negative correlation, while red signify a positive correlation. *, **, and *** denote p-value<0.05, p-value<0.01, and p-value<0.001.

Spearman’s correlation analysis explored the relationships between functional shifts and species abundances. The pathways responsible for LPS biosynthesis (PWY-7323 and OANTIGEN-PWY) and the degradation of SCFAs (ANAEROFRUCAT-PWY) were positively related to *Escherichia coli*, *Bifidobacterium longum*, *Clostridium bolteae*, *Bacteroides fragilis*, and *Klebsiella pneumonia* enriched in the older group (p-value<0.05, **Figure 5**). 

### Establishment and Verification of a Predictive Model of Age by the Gut Microbiota

To establish a simple non-invasive model of aging, 180 subjects (106:74) were included in a training dataset, while 434 subjects (266:168) were assigned to the test dataset. A multifactor logistic regression method was further used to establish the prediction model, which showed nine species (*Bacteroides fragilis*, *Barnesiella intestinihominis*, *Bifidobacterium longum*, *Clostridium bolteae*, *Escherichia coli*, *Klebsiella pneumoniae*, *Megamonas funiformis*, *Parabacteroides merdae*, and *Subdoligranulum unclassified*) were factors of aging in the training dataset. The area under the receiver operating curve (AUC) was 0.764 and 0.740 in training and test datasets, respectively (**Table 3**, **Supplemental Figure 1**).

**Table 3:** Model inclusion variables and logistic regression coefficients.

Index	β
*Bacteroides fragilis*	0.33
*Barnesiella intestinihominis*	-0.16
*Bifidobacterium longum*	0.20
*Clostridium bolteae*	1.82
*Escherichia coli*	0.08
*Klebsiella pneumoniae*	0.14
*Megamonas funiformis*	-0.19
*Parabacteroides merdae*	-0.29
*Subdoligranulum unclassified*	-0.14

Receiver operating characteristics (ROC) curves predict age in the training and test dataset. ROC (Bacteroides fragilis, Barnesiella intestinihominis, Bifidobacterium longum, Clostridium bolteae, Escherichia coli, Klebsiella pneumoniae, Megamonas funiformis, Parabacteroides merdae, and Subdoligranulum unclassified) based on multifactor logistic regression.

## Discussion

The current global population has entered an aging society. According to the United Nations Population Development Program, Chinese people over 65 years will reach 334 million in 2050, making it the country with the most significant number of older people in the world. China will face severe challenges of population aging in the future. Studies have shown that aging is now the most profound risk factor for almost all non-communicable diseases (including cardiovascular disease, cancer, diabetes, and neurological diseases) (Wagner et al., 2016) with the steady increase in older people. It was related to cellular aging, the accumulation of reactive oxygen species (ROS), damage to antioxidant defenses, mitochondrial dysfunction, DNA damage and telomere shortening, and so on (Park and Yeo, 2013). In addition to calorie restriction, SIRT1 activator resveratrol, mTORC1 complex inhibitor, telomerase activator TA-65, and other anti-aging treatments, probiotics also played a particular role in the fight against aging (Ros and Carrascosa, 2020). Significant life-extending effects of *Lactobacillus gasseriSBT2055* supplementation were repeatedly found in nematode Caenorhabditis elegans (Nakagawa et al., 2016). These pro-longevity effects were accompanied by stimulating the innate immune response signaling, improved resistance to oxidative stress, decreased lipofuscin accumulation, and modulated serotonin signaling (Grompone et al., 2012; Komura et al., 2013; Park et al., 2015). There was also evidence that *Bifidobacterium animalis subsp. lactis LKM512* could promote longevity in mice, possibly through suppressing chronic low-grade inflammatory processes in the colon (Matsumoto et al., 2011). Moreover, there was strong experimental and clinical evidence that probiotic supplementation may improve metabolic and cardiovascular health status by modulating crucial parameters in aging processes. Probiotic therapy lowered LDL levels, improved the low-density/high density lipoprotein ratio, and reduced inflammatory mediators, blood pressure, blood glucose levels, and BMI (Thushara et al., 2016). Our article aimed to analyze the alterations characteristics of intestinal microbes in the aging population and provided new ideas for delaying and anti-aging.

Our metagenomic study found that the following bacteria were enriched in the older group, *Clostridium bolteae, Escherichia coli, Escherichia unclassified, Parabacteroides distasonis, Parabacteroides unclassified*, and *Ruminococcus gnavus*. This is consistent with the results of previous studies (Salazar et al., 2017; Vaiserman et al., 2017; Kim and Jazwinski, 2018; Mangiola et al., 2018; Bana and Cabreiro, 2019; Badal et al., 2020; DeJong et al., 2020). These gut microbes were associated with increased chronic inflammation, including up-regulated synthesis of pro-inflammatory cytokines, such as interleukins (ILs)-6, -8, and -10, and tumor necrosis factor (TNF), thereby reducing activation of the level of lymphocytes, natural killer cells and phagocytic activity (Badal et al., 2020). This is consistent with the results of our study, which PWY-7323 and OANTIGEN-PWY involved in the production of LPS were enriched in the older group. *Escherichia coli, Bifidobacterium longum, Clostridium bolteae, Bacteroides fragilis*, and *Klebsiella pneumoniae* were positively correlated with PWY-7323 and OANTIGEN-PWY. Studies have mentioned that LPS will reduce mucin production, affect the balance of the mucus layer of the gastrointestinal barrier, increase the permeability of the intestine, and cause or aggravate inflammation (Bana and Cabreiro, 2019). Therefore, we considered that the bacteria enriched in the older group caused aging by mediating inflammation. LPS promoted proinflammatory cytokines and modulated signaling pathways involved in the pathogenesis of Alzheimer’s disease (Cattaneo et al., 2017), a disease with a higher prevalence rate among the elderly. Inflammation associated with aging can significantly impair brain function through increased expression of inflammatory cytokines and oxidative stress, destruction of the blood-brain barrier, infiltration of peripheral immune cells, and activation of glial cells (Cattaneo et al., 2017).

We also found that *Barnesiella intestinihominis* and *Subdoligranulum unclassified* are enriched in the younger group. In murine cancer models treated with cyclophosphamide, *Barnesiella intestinihominis* increased in abundance within the colon during treatment. *Barnesiella intestinihominis* in patients with colorectal cancer decreased and raised in control (Touchefeu et al., 2021). (Dizman et al., 2021) found that *Barnesiella intestinihominis* increased in patients with metastatic renal cell carcinoma treated with antitumor drugs (vascular endothelial growth factor tyrosine kinase inhibitor) after *Bifidobacterium*‐containing yogurt treatment. These patients also had more significant clinical benefits, such as decreased diarrhea severity. Besides, *Barnesiella intestinihominis* selectively predicted longer progression-free survival in advanced lung and ovarian cancer patients treated with chemo-immunotherapy (Daillere et al., 2016). These phenomena were considered that *Barnesiella intestinihominis* might have distinct immunomodulatory properties. The abundance of this bacteria correlated with several other immunoregulatory cells such as marginal zone B cells and invariant NKT in the spleen and liver. It significantly affected the abundance of Th1 and Tc1 cells in the multifunctional spleen, increased the recruitment or proliferation of IFN-g+ gdT cells in TIL (Dizman et al., 2021) (Touchefeu et al., 2021) (Daillere et al., 2016). It was worth mentioning that *Barnesiella intestinihominis* densities were abnormally elevated in HIV-infected compared with non-infected individuals and associated with systemic inflammation. Supporting this view, this bacterium was found overrepresented after ionizing radiation causing oxidative DNA damage, a therapeutic context where intestinal microbiota has a protective role. Except for *Barnesiella intestinihominis*, previous research found *Subdoligranulum unclassified* was positively correlated with CD8+HLA-DR+ T cell count in HIV infection, and further analysis showed that it had a close connection with the CD4 T-cell counts and T-cell immune activation (Lu et al., 2018). This indicated that changes of gut microbiota may be involved in immune activation. *Subdoligranulum unclassified* was thought to be beneficial for human health due to the fact that it has the ability of butyrate production (Louis and Flint, 2009; Lu et al., 2018). We analyzed the functional inheritance of bacteria and found that ANAEROFRUCAT-PWY was responsible for the degradation of SCFAs. *Barnesiella intestinihominisen*, *Parabacteroides merdae*, and *Megamonas funiformis* riched in the younger group were negatively related to ANAEROFRUCAT-PWY. Hence, we considered that the bacteria enriched in the younger group delayed aging by producing SCFAs. Studies have shown that SCFAs provide an energy source for colonic epithelial cells by activating intestinal gluconeogenesis; moreover, they can also improve inflammatory response, enhance immune regulation and increase anti-cancer ability by maintaining the intestinal barrier function, preventing metabolic endotoxemia, and enhancing mitochondrial activity (Vaiserman et al., 2017). Interestingly, adult flies fed sodium butyrate at concentrations of 10 and 20 mmol/l significantly prolonged the average life span of both males and females compared with the control group. Butyrate is known to inhibit histone deacetylase activity, thus inducing hyperacetylation of histones, hence tending to release histones from their binding to chromatin, with consequent effects on gene transcription (Kang et al., 2002). Taken together, we speculated that metabolites produced by gut microbes could delay aging by participating in immune regulation, anti-inflammatory, modulating epigenetic processes by inhibition of histone deacetylase activity.

In previous studies, the elderly was defined as over 60 years old (WHO defines seniors as 60 years old and above) or more than 65 decades old. We chose the 50-year-old as the demarcation point for grouping, which was different from previous studies. On the one hand, further animal studies have shown gender differences in the gut microbiota of adult mice (Org et al., 2016) and pigs (He et al., 2019), emphasizing the critical role of sex hormones. On the other hand, women’s gut microbes before and after menopause (aged 50) have also changed (Zhao et al., 2019); in addition, testosterone levels in men gradually decrease with age (32-44 years) (Dabbs, 1990). Our research also has limitations, such as not detecting inflammatory factors and bacterial metabolites and not detecting hormones in the included population.

## Conclusions

This study found extensive and interesting changes in association with the gut microbiota composition and functions with aging in China, which were characterized by increased levels of *Bacteroides fragilis, Bifidobacterium longum, Clostridium bolteae, Escherichia coli, Klebsiella pneumoniae*, decreased levels of *Megamonas funiformis, Parabacteroides merdae, Barnesiella intestinihominis* and *Subdoligranulum unclassified*. The degradation of SCFAs and the production of LPS are all closely related to aging. In addition, AUC indicates that supplementing with probiotics (*Barnesiella intestinihominis* and *Subdoligranulum unclassified*) may affect aging. The gut microbiota communicates with the host through various biomolecules, nutrient signaling-independent pathways, and epigenetic mechanisms. Interference with these communications by age-related alterations in the gut microbiota can affect the host’s health. Although further studies are necessary to validate the function of microbiota between groups, this study provides valuable information on aging’ gut microbiota.

## Data Availability Statement

The datasets presented in this study can be found in online repositories. The names of the repository/repositories and accession number(s) can be found below: https://www.ebi.ac.uk/ena, PRJEB53016.

## Ethics Statement

The studies involving human participants were reviewed and approved by The studies involving human participants were reviewed and approved by an ethics committee from the First Affiliated Hospital of Zhengzhou University (Approval number: 2018-KY-56). The patients/participants provided their written informed consent to participate in this study.

## Author Contributions

HY and QQ wrote and edited the manuscript, contributing equally to this study. SY and JC performed data analysis. TL, XG, and YY helped to collect samples and data. SD was in charge of the project. All authors have read and agreed to this version of the manuscript.

## Funding

The study was supported by the National Natural Science Foundation of China (72101236), Henan Province Key Specialized Research and Development Breakthrough Projects (222102310226), Henan Province Key Scientific Research Projects of Universities (21A320035), Youth Talent Promotion Project of Henan Province (2021HYTP052), Henan Province Medical Science and Technology Research Plan (LHGJ20200279), and Chinese National Science and Technology Major Project (2018ZX10305410).

## Conflict of Interest

The authors declare that the research was conducted without any commercial or financial relationships that could be construed as a potential conflict of interest.

## Publisher’s Note

All claims expressed in this article are solely those of the authors and do not necessarily represent those of their affiliated organizations, or those of the publisher, the editors and the reviewers. Any product that may be evaluated in this article, or claim that may be made by its manufacturer, is not guaranteed or endorsed by the publisher.
