# Supplementary figures and images for: Comparison Of The Gut Microbiota In Different Age Groups In China
Source: Front Cell Infect Microbiol. 2022 Jul 25;12:877914. doi: 10.3389/fcimb.2022.877914 (PMC9359670; doi:10.3389/fcimb.2022.877914)

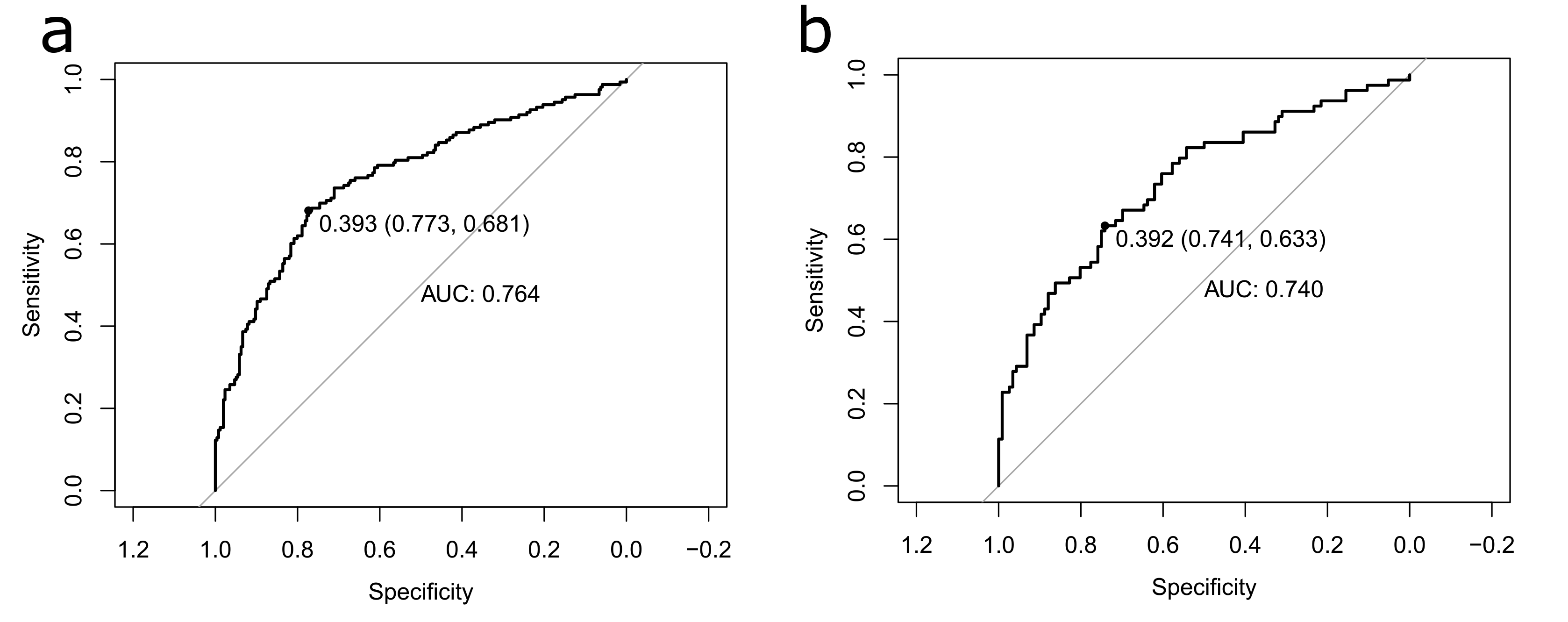

Supplement: Supplementary file 1 [file Image_1.tif]
